# Supplementation with α-Lipoic Acid, CoQ10, and Vitamin E Augments Running Performance and Mitochondrial Function in Female Mice

**DOI:** 10.1371/journal.pone.0060722

**Published:** 2013-04-02

**Authors:** Arkan Abadi, Justin D. Crane, Daniel Ogborn, Bart Hettinga, Mahmood Akhtar, Andrew Stokl, Lauren MacNeil, Adeel Safdar, Mark Tarnopolsky

**Affiliations:** Department of Pediatrics, McMaster University, Hamilton, Ontario, Canada; Bambino GesùChildren Hospital, Italy

## Abstract

Antioxidant supplements are widely consumed by the general public; however, their effects of on exercise performance are controversial. The aim of this study was to examine the effects of an antioxidant cocktail (α-lipoic acid, vitamin E and coenzyme Q10) on exercise performance, muscle function and training adaptations in mice. C57Bl/J6 mice were placed on antioxidant supplement or placebo-control diets (n = 36/group) and divided into trained (8 wks treadmill running) (n = 12/group) and untrained groups (n = 24/group). Antioxidant supplementation had no effect on the running performance of trained mice nor did it affect training adaptations; however, untrained female mice that received antioxidants performed significantly better than placebo-control mice (p ≤ 0.05). Furthermore, antioxidant-supplemented females (untrained) showed elevated respiratory capacity in freshly excised muscle fibers (*quadriceps femoris*) (p ≤ 0.05), reduced oxidative damage to muscle proteins (p ≤ 0.05), and increased expression of mitochondrial proteins (p ≤ 0.05) compared to placebo-controls. These changes were attributed to increased expression of proliferator-activated receptor gamma coactivator 1α (PGC-1α) (p ≤ 0.05) via activation of AMP-activated protein kinase (AMPK) (p ≤ 0.05) by antioxidant supplementation. Overall, these results indicate that this antioxidant supplement exerts gender specific effects; augmenting performance and mitochondrial function in untrained females, but does not attenuate training adaptations.

## Introduction

Dietary antioxidant supplements are readily available and routinely consumed by the general public. Reasons for supplementation include compensation for dietary insufficiencies, treatment of primary mitochondrial disorders, improved health and overall wellbeing, and the prevention of diseases associated with aging including cancer. Despite estimates that 40% of the adult population in the U.S consume some form of antioxidants [1], scientific support for their purported benefits is currently inadequate and some reports even suggest that antioxidants may be detrimental to some health outcomes [2], [3], [4]. Exercise is believed to result in a transient increase in reactive oxygen species (ROS) production that results from increased demand for ATP, produced by mitochondrial oxidative phosphorylation, to support increased muscle contractile activity [5], [6]. This increase in ROS may play a role in activating signaling pathways that mediate long-term adaptations to exercise, such as increased mitochondrial biogenesis [6], that is associated with improved insulin sensitivity [7]. Conversely, excessive ROS production may place muscle in a condition of oxidative stress that adversely affects performance and adaptation [8]. Consequently, the hypothesis has emerged that the consumption of antioxidants during endurance training could attenuate physiological adaptations in muscle (increased antioxidant defense enzyme activity and mitochondrial biogenesis). The latter concept has been controversial since some reports have shown that antioxidant supplementation counteracted the beneficial effects of exercise, reduced performance, and inhibited mitochondrial adaptations to exercise [9], [10], but others have not [11], [12], [13], [14].

Important factors that may contribute to differences between reported outcomes include; the complex interactions between antioxidants, the potential for antioxidants to become pro-oxidant, non-redox cellular effects of the “antioxidants”, and/or temporal differences between the state of training (early adaptive or elite acute extreme performance). Although there are many different compounds that fall under the umbrella term “antioxidant”, vitamin E, vitamin C, coenzyme Q_10_ (CoQ_10_) and α-lipoic acid are more commonly studied. CoQ_10_ is an electron-shuttling compound that is vital to the mitochondrial electron transport chain and has potent antioxidant properties [15]. Vitamin E (α-tocopherol) is an important component of biological membranes where it is thought to participate in scavenging lipid peroxide radicals [16], [17], though it may exert some molecular effects independent of its antioxidant properties [18], [19]. α-Lipoic acid is an essential cofactor for mitochondrial α-ketoacid dehydrogenases (e.g. pyruvate dehydrogenase) and is crucial for several mitochondrial metabolic pathways [20], [21]. It is also regarded as potent mitochondrial antioxidant and has been studied in numerous oxidative stress-related pathological conditions such as ischemia/reperfusion injury and diabetes [22], [23]. It also has direct effects on skeletal muscle, increasing resting energy expenditure, insulin sensitivity, and glucose uptake [23], [24]. This is mediated primarily through the activation of AMP-activated protein kinase (AMPK), a major energy (ATP/AMP) sensor in the cell [24], [25], that regulates mitochondrial biogenesis through peroxisome proliferator-activated receptor gamma coactivator 1α (PGC-1α) [26], [27].

Despite the potential effects of these and other antioxidant compounds on metabolism and energetic adaptation, there is no evidence that mitochondrial function can be directly altered by dietary antioxidant supplementation alone. While many of these antioxidants display potent radical scavenging properties *in vitro*, experiments conducted *in vivo* are mixed and some studies indicate that they may function as pro-oxidants, rather than antioxidants [28], [29], [30], [31]. This study was undertaken to investigate the effects of dietary supplementation with vitamin E, α-lipoic acid, and CoQ_10_ in combination on basal and training-induced mitochondrial adaptations in mice. These compounds were specifically selected because they are used in the treatment of mitochondrial cytopathies and have been shown to reduce oxidative stress and enhance mitochondrial function in humans [32], [33], [34]. In addition to patients with primary genetic mitochondrial myopathies, the role for mitochondrial dysfunction in many disorders associated with human aging further strengthens the need to understand the potential role of antioxidants as a therapeutic intervention.

## Results

**Figure 1:**
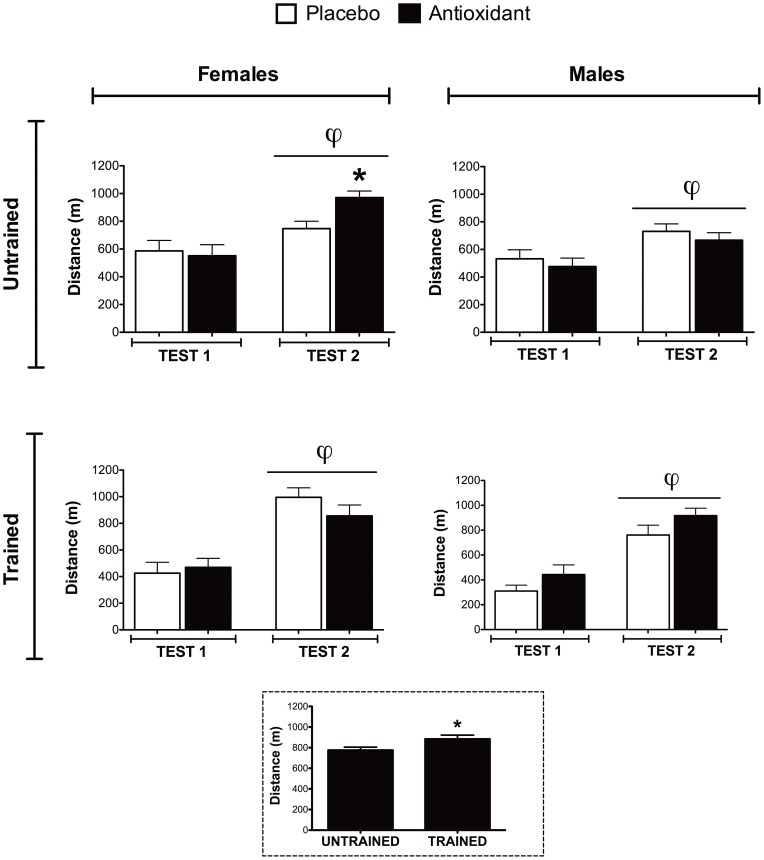
Exercise performance testing. Performance tests were conducted at the beginning (Test 1) and end of the study (Test 2). Running distance, calculated from both treadmill speed increments and time till exhaustion, was plotted. The performance of all the groups improved over the course of the study (Test 1 vs. Test 2, φ indicates main effect of testing, p≤0.05). Post-hoc testing was used to compare antioxidant- and placebo-supplemented groups, * indicates p≤0.05. Effect of training on performance is shown in the inset figure (dotted border) represented by the distance run during Test 2 for trained and untrained mice (unpaired T-Test, * indicates p≤0.05).

All mice completed two performance tests, Test 1 occurred at the beginning of the study, and Test 2 after 7 wk of antioxidant supplementation (or placebo) with and without training. All groups of mice showed significant improvements in exercise performance (distance run) between Test 1 and Test 2, which is likely due to growth and maturation over the course of the study (Figure 1, p < 0.01). Female mice that received antioxidant supplements but no training (untrained) improved running performance (distance run) (Figure 1) to a greater extent (p < 0.05) than their placebo-fed counterparts. This performance effect of antioxidant supplementation was not seen for the untrained male mice (Figure 1). Antioxidant supplementation did not differentially alter the performance of trained male or female mice and, as expected, the trained mice performed significantly better (distance run, p < 0.01) than untrained mice (Figure 1 inset). Thus, antioxidants appear to exert sex specific benefits in untrained female mice but did not affect training-induced improvements in performance in either male or female mice.

**Figure 2:**
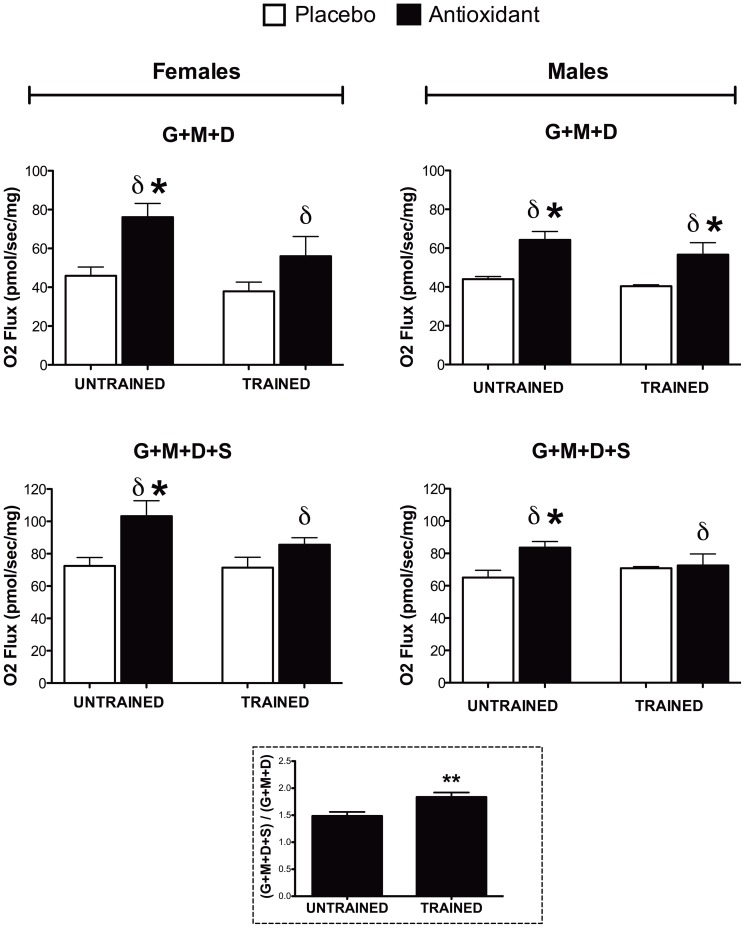
Mitochondrial respiratory capacity. Respiratory capacity was assessed in permeabilized fibers from *quadriceps femoris* muscle tissue (n = 3–4). Maximal coupled respiration was measured in the presence of complex I substrates (glutamate and malate) and ADP (G+M+D, upper panels) followed by addition of the complex II substrate succinate to measure maximal coupled respiration through both complex I + II (G+M+D+S, lower panels). δ indicates main effect of antioxidant supplementation (p≤0.05), and post-hoc testing was used to compare antioxidant- and placebo-supplemented groups, * indicates p≤0.05. Effect of training on performance is shown in the inset figure (dotted border) as represented by the ratio of complex I+II respiration (G+M+D+S) to complex I respiration (G+M+D) (unpaired T-Test, ** indicates p<0.01).

As the reliability of performance tests in mice have been criticized [35], [36], experiments were conducted to ascertain whether antioxidant supplementation produced biochemical and molecular alterations in muscle. To this end, the maximal coupled mitochondrial respiratory capacity in freshly excised fibers of *quadriceps femoris* muscle from these mice was determined. Measurements were performed on permeabilized fibers using complex I respiratory substrates (glutamate/malate + ADP) and then succinate was added to obtain maximal complex I + II respiration (glutamate/malate + ADP + succinate) (Figure 2). Antioxidant supplementation was associated with higher maximal respiration relative to placebo-fed mice as a main effect for antioxidant supplement (δ, p < 0.05, Figure 2). Post-hoc testing showed that the antioxidant supplement only increased respiratory capacity for the females in the untrained state and for the males in all training states except for the G + M + D + S for the trained group (p < 0.05, Figure 2). The RCR (State 3/State4) was lower in the trained animals with this reduction being prevented for the female animals on antioxidant supplement (P< 0.05, Figure S7). Basal respiration (State 4 surrogate) was not significantly altered by training or antioxidant supplement (Figure S7). Somewhat unexpectedly, training *per se* did not lead to significantly higher maximal respiratory capacity. However, similar results were reported by another group which showed that training did not increase maximal respiration stimulated by complex I substrates (pyruvate + ADP) but significantly increased maximal respiration with complex II substrates (succinate + ADP)[37]. Consequently, we calculated the proportion of maximal respiration from complex II (G+M+D+S / G+M+D), which is a crude estimate of complex II respiratory capacity, and found a significant (p < 0.01) training-induced increase in respiratory capacity through complex II (Figure 2, inset). Another factor that likely explains the lack of a training effect in this data set is the fact that the respiratory capacity measurements were completed in the *quadriceps* muscle and subsequent data from our laboratory has found that exercise adaptations in this muscle are less robust than in the *tibialis anterior* muscle (see below) when mice run on a level treadmill (as opposed to an inclined treadmill)(Crane, et al, unpublished observations, 2012). These latter findings likely attenuated our ability to detect a training effect upon this outcome metric but would not have altered our conclusions regarding the effect of the antioxidant *per se* on the respiratory capacity of the muscle.

**Figure 3:**
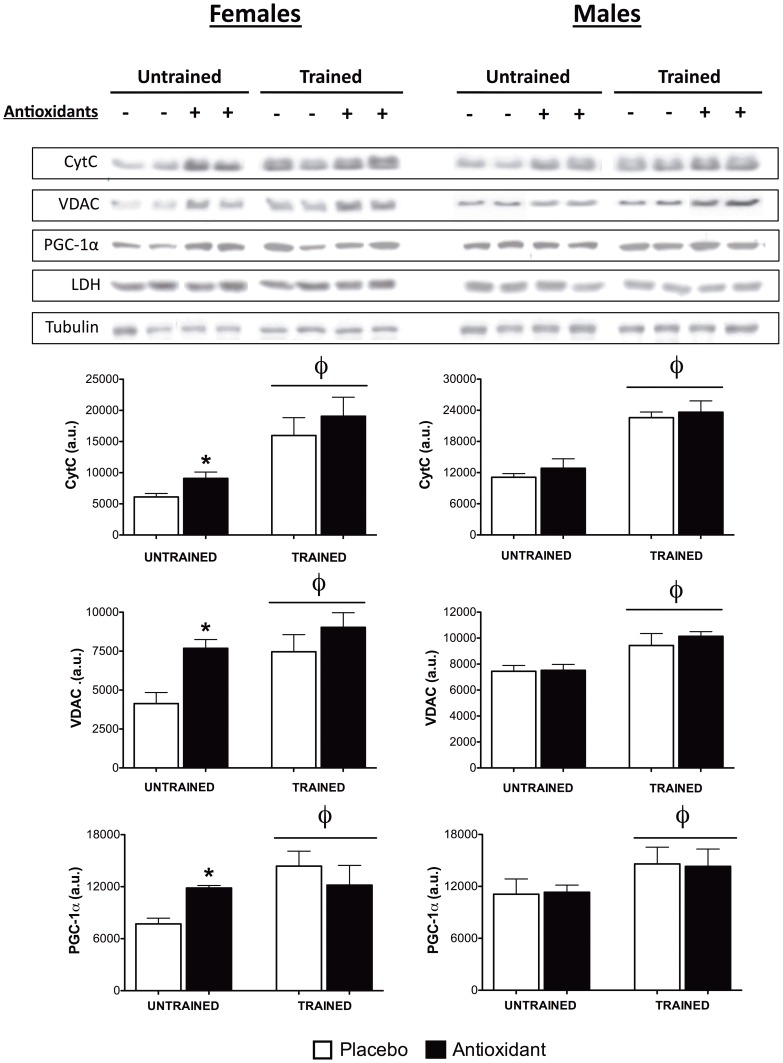
Expression of mitochondrial proteins and PGC-1α. Expression of mitochondrial proteins and PGC-1α were determined using Western blotting on protein homogenates prepared from *tibialis anterior* muscle tissue (n = 4–6 mice/gender/group). Data are means ± SEM and representative bands from each gender and group are shown. φ indicates main effect of training, p≤0.05, and post-hoc testing was used to compare antioxidant- and placebo-supplemented groups, * indicates p≤0.05.

Next, the expression of several mitochondrial proteins in homogenates prepared from skeletal muscle was examined using Western blotting. In female mice, training was associated with higher protein content (p < 0.01) for; cytochrome C (cytC), voltage dependent anion channel (VDAC), as well as subunits within complex I, II, III, IV and V of the mitochondrial electron transport chain (Figure 3, S1 and S2). Post-hoc testing indicated that the protein content of cytC, VDAC, complex III, and complex IV was significantly (p < 0.05) higher in antioxidant-supplemented untrained females, as compared to placebo-treated mice (Figure 3 and S1). Training was also associated with significantly (p < 0.01) higher protein content of cytC, VDAC, complex II, and III in male mice, but the expression of these proteins was not differentially affected by antioxidant supplementation (Figure 3, S1, and S2). PGC-1α protein content was also significantly higher in trained mice (p < 0.05) for both males and females (Figure 3). Furthermore, antioxidant supplementation was associated with significantly higher PGC-1α protein content in untrained females (p < 0.05), but had no differential effect on PGC-1α following training (Figure 3). Mitochondrial transcription factor A (Tfam), was similarly higher (p < 0.05) in untrained females that were supplemented with antioxidants (Figure S1). The aforementioned results suggest that the supplementation-associated improvements in performance, respiratory capacity, and mitochondrial protein expression in untrained female mice were associated with higher PGC-1α protein content and that the expected training induced adaptations to exercise are manifest in the *tibialis anterior* muscle during level treadmill endurance running.

**Figure 4:**
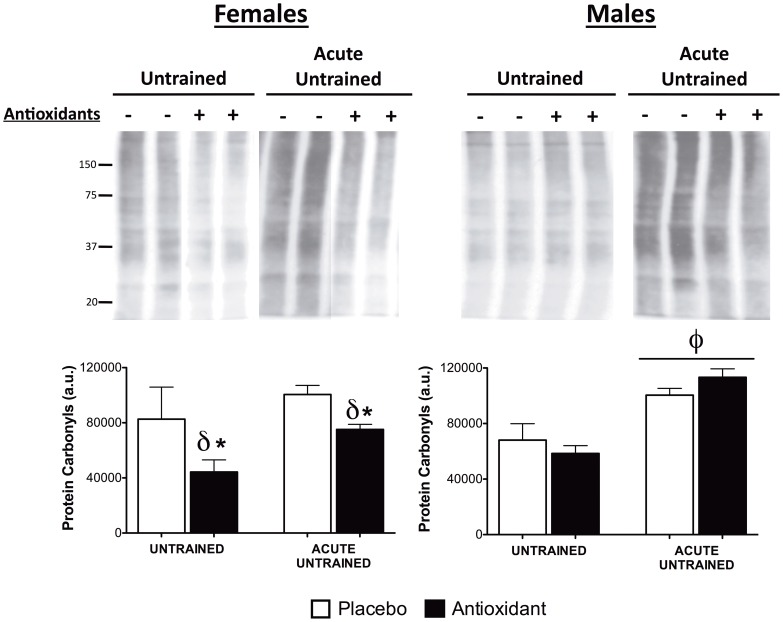
Oxidative damage to proteins. Oxidative damage to proteins was assessed using anti-protein carbonyl antibody, and whole lane staining was quantified as in Figure 3. φ indicates a significant (p≤0.05) main effect of exercise, δ indicates a significant (p≤0.05) main effect of antioxidant supplementation, and post-hoc testing was used to compare antioxidant- and placebo-supplemented groups, * indicates p≤0.05.

The most plausible mechanism by which antioxidant supplements function is by reducing oxidative stress, quenching ROS generated by metabolic processes, and sparing essential cellular molecules from free radical damage. To determine whether antioxidant supplementation and/or training altered antioxidant defense enzymes in skeletal muscle, the protein content of manganese superoxide dismutase (MnSOD), copper/zinc SOD (Cu/ZnSOD) and catalase was assessed. Cu/ZnSOD and catalase protein levels were not affected by antioxidant supplementation or training (Figure S3); however, MnSOD protein was significantly higher after training (p < 0.01), and MnSOD was significantly higher (p < 0.05) in antioxidant supplemented untrained female mice (Figure S3). In addition, we also evaluated mRNA abundance of Cu/ZnSOD (SOD1), MnSOD (SOD2) and cyclooxygenase and found no acute effect of exercise or antioxidant effect (Figure S6). Next, the level of protein carbonyls, a marker of oxidative damage to proteins, was evaluated in skeletal muscle homogenates from untrained mice at rest and following an acute bout exhaustive exercise. In females, antioxidant supplementation was associated with significantly (p < 0.05) lower levels of protein carbonyls in untrained mice both at rest and following acute exercise (Figure 4). In males, acute exercise significantly increased protein carbonyl levels (p < 0.05) on protein carbonyl levels, but this was unaffected by antioxidant supplementation (Figure 4). These results indicate antioxidant supplementation may protect cellular proteins from exercise induced oxidative damage by directly quenching ROS in female mice and not by altering protein content of important antioxidants.

**Figure 5:**
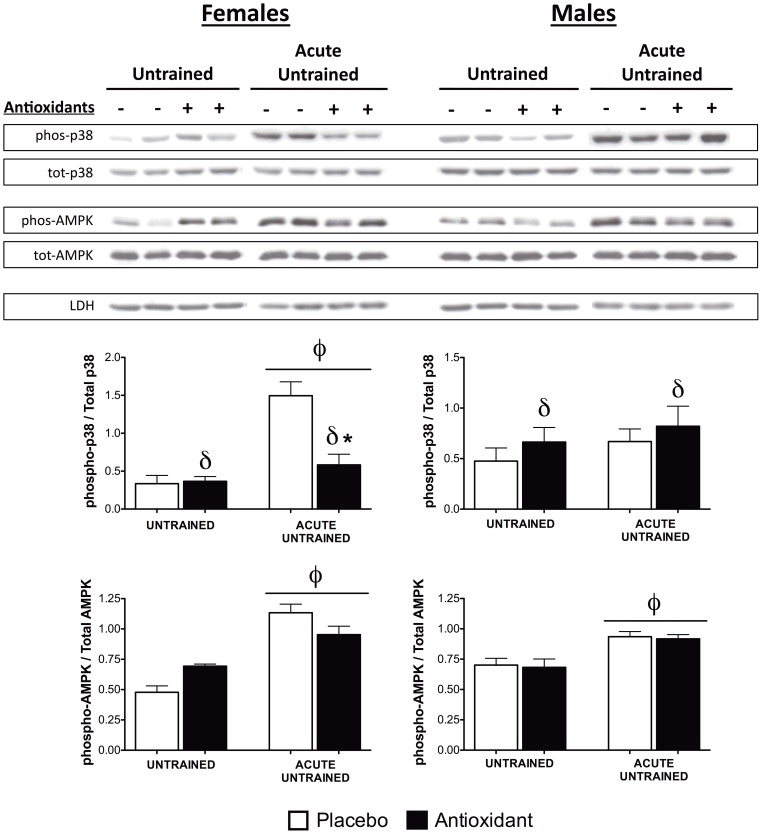
Activation of signaling kinases. Protein expression of total-p38 MAPK, phospho-p38 MAPK (Thr180/Tyr182), total-AMPK, and phospho-AMPK (Thr172) was determined as in Figure 3. The ratio of phosphorylated bands to total bands was determined and plotted. φ indicates a significant (p≤0.05) main effect of exercise, δ indicates a significant (p≤0.05) main effect of antioxidant supplementation, and post-hoc testing was used to compare antioxidant- and placebo-supplemented groups, * indicates p≤0.05.

In addition, several signaling pathways that are relevant to exercise adaptations and oxidative stress were examined to identify specific pathways that may respond to antioxidant supplementation. AMPK is thought to play a crucial role in cellular energy homeostasis and can activate PGC-1α to induce mitochondrial biogenesis [27], [38]. AMPK phosphorylation was significantly higher following an acute bout of exercise in untrained mice (p < 0.01, Figure 5) and basal AMPK phosphorylation was significantly higher with antioxidant supplementation in untrained females (p < 0.05). The effects of antioxidant supplementation on the activation of stress kinases in the mitogen-activated protein kinase (MAPK) pathway were also examined as these pathways are involved in several exercise-induced adaptations [39], [40], [41]. Phosphorylation of both extracellular-signal-regulated kinase (ERK) 1/2 and c-Jun N-terminal kinase (JNK) 1/2 was significantly higher following acute exercise in untrained mice (p < 0.05), but was unaffected by antioxidant supplementation (Figure S4). On the other hand, phosphorylation of p38-MAPK differed in male and female mice. In untrained females, phosphorylation of p38-MAPK was significantly higher (p < 0.05) following an acute bout of exercise, and *post-hoc* testing indicated that this increase was markedly suppressed (p < 0.01) by antioxidant supplementation (Figure 5). In untrained males, antioxidant supplementation was associated with significantly greater p38-MAPK activation (p < 0.05, Figure 5). These results indicate that activation of p38-MAPK following acute exercise in untrained females is specifically suppressed by antioxidant supplementation, while antioxidant supplementation is generally associated with increased activation of p38-MAPK in males.

## Discussion

The overall findings of this study are three fold. First, supplementation with α-lipoic acid, vitamin E, and CoQ_10_ improved exercise performance and mitochondrial respiratory chain protein capacity in untrained female mice. Second, these antioxidants had no deleterious effect on the training-induced adaptations for either male or female mice. Finally, the marked sex differences in several of the outcomes highlights the importance of considering the influence of sex upon outcome measures in studies evaluating exercise and nutritional interventions.

Mitochondrial biogenesis is a tightly controlled process in which cellular energy demands are balanced against ROS production and oxidative stress. Antioxidant supplementation may shift this balance in favor of increased mitochondrial biogenesis by directly scavenging radicals or boosting endogenous antioxidant defenses. Results herein indicate that the level of protein carbonyls, a known marker of oxidative damage to proteins, was significantly lower after antioxidant supplementation in untrained female mice; however the expression of several antioxidant defense enzymes was not significantly altered. It is therefore likely that the antioxidant supplement used here acted directly to protect cellular proteins from oxidative damage, perhaps by scavenging ROS. Although antioxidant supplementation may indirectly support increased mitochondrial content, mitochondrial biogenesis requires the activation of several other regulatory pathways.

Signaling pathways that regulate mitochondrial biogenesis have been extensively investigated over the past decade and several pathways have been uncovered that culminate in the activation of PGC-1α, a master regulator of mitochondrial biogenesis [42]. AMPK is an important regulator of energy homeostasis that is activated by declines in the ratio of ATP:AMP to stimulate translocation of GLUT-4, increase glucose uptake, and stimulate mitochondrial biogenesis to restore the ATP:AMP ratio [27], [38], [43], [44]. AMPK has been shown to activate mitochondrial biogenesis through activation of PGC-1α [24], [45], [46]. Importantly, α-lipoic acid is known to activate AMPK and has been effectively used to increase insulin sensitivity in type 2 diabetic patients [23], [25]. Although the precise mechanism by which α-lipoic acid activates AMPK is not fully understood, there is some evidence that it is dependent on the activation of CAMK in skeletal muscle [47]. α-Lipoic acid may also modulate intracellular signaling pathways independent of its antioxidant properties. It has been shown to inhibit inhibitor of nuclear factor kappa-B kinase subunit beta (IKK2), an inhibitor of nuclear factor kappa-light-chain-enhancer of activated β cells (NF-κB), leading to the activation of NF-κB-dependent gene transcription [48]. Also, α-lipoic acid has been reported to decrease food intake and body weight while increasing ambulatory movement and physical activity in mice, which may further stimulate mitochondrial function in skeletal muscle [24]. Since α-lipoic acid is a component of the antioxidant supplement used here, AMPK activation is an excellent mechanistic candidate for the effects of antioxidant supplementation described above and its activation in untrained female mice supplemented with antioxidants was confirmed. However, antioxidant supplementation was not associated with decreased food intake or body weight in this study (Figure S5), indicating that it did not act as an anorexic nor is it likely to have increased ambulatory movement. The cause of this discrepancy is unclear though it is possible that supplementation vitamin E and CoQ_10_ counteracted the potential anorexic effects of α-lipoic acid since rats receiving long-term supplementation with CoQ_10_ have higher body weights when compared to placebo-treated controls [49]. Additionally, trained rats receiving a mitochondrial nutrient cocktail that included α-lipoic acid and CoQ_10_ (12 wks) did not show any weight loss when compared to controls [50].

The MAPK pathway is activated during cell stress in numerous contexts. This pathway consists of three distinct but overlapping branches including p38-MAPK, ERK 1/2, and JNK 1/2 pathways. These branches have all been shown to respond to acute exercise in skeletal muscle but the extent of their involvement appears to depend on the specifics of the exercise stimulus (intensity and duration) [39]. ROS can activate the MAPK pathway directly, through redox sensitive thiol groups in the upstream kinase apoptosis signal-regulating kinase 1 (ASK1) which regulates p38-MAPK and JNK 1/2, or indirectly via release of calcium stores that regulate ERK 1/2 and JNK 1/2 [51], [52], [53]. Activation of p38-MAPK has been implicated in the activation of PGC-1α following exercise either through its down-stream target, activating transcription factor 2 (ATF2) [40] or through direct phosphorylation of PGC-1α [41]. ERK 1/2 can induce the activation of NF-κB, via mitogen- and stress-activated protein kinase (MSK1)[52], [53]. NF-κB is an important mediator of cellular responses to oxidative stress and its activation results in the induction of antioxidant defense genes that contain antioxidant response element (ARE) sequences within their promoter regions [54]. For example, supplementation with vitamin E, C, and α-lipoic acid was shown to inhibit the activation of JNK in skeletal muscle by high fat feeding in rats [55].

At rest there was no difference in the activation of p38-MAPK, ERK 1/2, or JNK 1/2 between antioxidant and placebo-supplemented untrained female mice, suggesting that these pathways do not participate in the enhanced exercise performance, respiratory capacity, and mitochondrial biogenesis in these mice. Acute exercise lead to increased activation of p38-MAPK, ERK 1/2, and JNK 1/2 in untrained mice, but only p38-MAPK was affected by antioxidant supplementation. In females, antioxidant supplementation markedly suppressed the activation of p38-MAPK by acute exercise, but in males, antioxidant supplementation was associated with greater p38-MAPK. These latter findings suggest that antioxidant supplementation specifically suppressed p38-MAPK activation following acute exercise in female mice, though this did not affect long-term adaptation to training. This is consistent with lower protein carbonyl levels seen in acutely exercised untrained female mice, as activation of p38-MAPK is regulated directly by ROS via ASK1 [51]. Of note, several gene targets of NF-κB, including cyclooxygenase2, sod1 and sod2, were examined using quantitative RT-PCR and the expression of these genes was not affected by antioxidant supplementation (Figure S6). This suggested that the NF-κB pathway was not modulated by the antioxidant supplement used in the current study. Overall, these findings suggest that the antioxidant supplement used here induced the activation of AMPK in the skeletal muscle of untrained female mice, which in turn activated PGC-1α, increased mitochondrial biogenesis (an total mitochondrial protein content) and improved performance. Antioxidant supplementation also reduced the activation of the stress kinase p38-MAPK following acute exercise in untrained female mice, which may improve tolerance to exhaustive exercise.

Antioxidant supplementation had little effect in male mice and most of the effects on performance, mitochondrial function and content, and signaling pathways were observed only in female mice. The physiological and molecular basis of this sex specificity is not clear, as little is known about how metabolic and signaling pathways are differentially regulated between sexes. Such differences are likely to be subtle and the sensitivity of current methodologies may not be sufficient to resolve them satisfactorily. In addition, most studies aimed at investigating the effects of antioxidant supplementation have been conducted in men, male rodents, or *in vitro* cell culture models and effects specific to females, as described here, may have been missed [9], [10], [12]. Not withstanding these issues, many of the differences between sexes are rooted in the function of hormone-responsive signaling proteins such as the androgen receptor and estrogen receptors. Interestingly, variants of vitamin E have been shown to bind to estrogen receptor, and some evidence suggests that specific isoforms of the estrogen receptor may translocate to mitochondria and regulate mitochondrial protein folding in response to mitochondrial stress [56], [57], [58]. Further experimentation using oophorectomy and estrogen supplementation are needed to determine whether modulation of estrogen receptor function by the antioxidant supplement used here may improve mitochondrial function and performance in untrained female mice.

The effects of antioxidant supplementation on exercise adaptation and training has been the subject of several recent investigations. Data from a similar training study in both humans (men) and rodents (male rats) found that vitamin C supplementation suppressed the effects of training on performance, expression of mitochondrial proteins (cytC) and regulators of mitochondrial biogenesis (e.g. PGC-1α) [9]. Another study in male rats indicated that combined supplementation with vitamin E and α-lipoic acid decreased PGC-1α mRNA, COXIV protein (but not cytC), in both trained and untrained animals [10]. On the other hand, Yfanti et al. found that combined supplementation with vitamin E and C in men resulted in increased expression of PGC-1α and peroxisome proliferator-activated receptor gamma (PPARγ) mRNA following 12 wk of endurance training as compared to placebo [12]. Another study demonstrated that combined vitamin E and C supplementation reduced chronic loading-induced markers of oxidative damage in young and old rats, improved antioxidant defenses, and resulted in improved power output in old rats [59]. Additionally, a similar study in rats found that vitamin E and C supplementation had no effect on endurance training-induced increases in PGC-1α and mitochondrial proteins, but did suppress exercise-induced increases in plasma TBARS [11]. Although research involving the effects of various antioxidants has made definitive conclusions difficult, the results presented herein are congruent with those indicating that antioxidant supplementation does not counteract the effects of exercise. Importantly, the novel finding that supplementation with α-lipoic acid, vitamin E and CoQ_10_ improved the performance and mitochondrial function in untrained female mice has not been previously reported and could have implications for the treatment of mitochondrial disorders.

While differences between the specifics of these studies, such as the dosage and duration of supplementation and training may contribute to differences between results reported here and those in the literature, it is also possible that differences in the composition of these supplements played a role. It is worth noting that CoQ_10_ supplementation in mice has been shown to increase both cytosolic and mitochondrial levels of vitamin E and CoQ_10_ in heart and skeletal muscle [49], [60], and α-lipoic acid supplementation significantly increased uptake of vitamin E in soleus of rats [61]. Furthermore, CoQ_10_ and α-lipoic acid have been shown to contribute to the recycling of α-tocopherol (vitamin E) from oxidized to reduced forms in isolated mitochondria [22], [62]. Vitamin E together with CoQ_10_ cooperatively protected serum from exogenous pro-oxidants [63], and CoQ_10_ supplementation also resulted in reduced levels of mitochondrial protein carbonyls in rat skeletal muscle [49]. These observations support the notion that vitamin E, α-lipoic acid, and CoQ_10_ supplementation may function cooperatively in a manner that may produce different outcomes from those reported using these antioxidants individually or in differing combinations.

Finally, it was of interest that the only outcome metric similar for both males and females was the improvement in mitochondrial respiratory capacity seen with both complex I and II substrates following antioxidant supplementation in both trained and untrained animals (main effect for supplementation) in the relatively untrained *quadriceps* muscle. Of interest, a short-term supplementation study in patients with mitochondrial disease (MELAS syndrome) found that a combination of α-lipoic acid, CoQ_10_, vitamin E and creatine monohydrate lowered plasma lactate levels [32], [33], [34]; a finding potentially associated with enhanced mitochondrial capacity. The finding of enhanced respiratory capacity was not associated with a consistent/correlative increase in total mitochondrial protein content; consequently, the mechanism behind the observation remains speculative, and further studies are required and should consider possibilities such as oxidative damage to the respiratory chain protein sub-units, proton leak, and possibly phosphorylation status of mitochondrial proteins (i.e., complex I).

In summary, these findings demonstrate that an antioxidant combination can augment mitochondrial content and function in skeletal muscle of female mice independent of an exercise intervention and that antioxidants did not attenuate many of the classical muscle adaptations to endurance exercise training. The unexpected finding of an increase in complex I and II respiratory capacity in both male and female mice following antioxidant supplementation is worthy of further investigation buy may have widespread implications in the treatment of a host of metabolic disorders including mitochondrial myopathies and insulin resistance. Although the effects of exercise *per se* are generally more robust and consistently reported, the mitochondrial effects reported herein from the antioxidant could be a useful therapeutic strategy in clinical populations that cannot exercise.

## Materials and Methods

### Ethics Statement

All procedures and protocols where approved by Animal Research Ethics Board at McMaster University and strictly adhere to the principles and policies of the Canadian Council on Animal Care (CCAC), and legislation as presented in the Animals for Research Act, Ontario (1980) and administered by the Ontario Ministry of Agriculture and Food (OMAF).

### Animals

C57Bl/J6 mice were obtained from Jackson Labs. All mice were housed in the Central Animal Facility at McMaster University on a 12h light/dark cycle, and given water and a standard chow diet (Harlan Teklad 8640 22/5 rodent diet) *ad libitum*.

### Study Design

A nested study design was implemented in which 72 age- and sex-matched mice were divided into two nutritional groups (antioxidant and placebo, n = 36/group) and then two exercise groups (trained n = 12/group, and untrained n = 24/group). All mice performed an exhaustive bout of exercise (Test 1) to establish baseline exercise performance measures (see below). Mice in the antioxidant group then received standard chow supplemented (custom diet, Harlan Teklad, Wisconsin, USA) with vitamin E (α-tocopherol, 1000 IU, Harlan Teklad, Wisconsin, USA), 0.1% α-lipoic acid (NatureGen Inc., California, USA), and 0.25% CoQ_10_ (Sigma-Aldrich, Missouri, USA), and mice in the placebo group received standard chow feed (Harlan Teklad 8640 22/5 rodent diet). After one week of acclimation, mice in the trained groups were entered into a 7-week exercise training program, detailed below, while mice in the untrained group did not receive any exercise. At the end of the training program, all mice were subjected to a second exhaustive bout of exercise (Test 2) to evaluate the effect of nutritional supplementation and training on exercise performance. A subset of mice in the untrained groups (n = 12/group) were sacrificed 3 h following performance Test 2 to evaluate the effects of 7 weeks of supplementation on the acute response to exercise in untrained mice. The remaining mice were restored to their respective nutritional supplementation and sacrificed 2 weeks after Test 2 to evaluate the effects of supplementation in untrained mice at rest. Mice in the trained groups resumed training for an additional week (total of 8 weeks) and were sacrificed 24 h after their final training session. All mice were sacrificed by cervical dislocation and skeletal muscle tissue was harvested and snap frozen in liquid nitrogen. Tissue was stored at –80°C until further analysis.

### Training and performance testing

Performance testing was conducted on a motorized 6 lane variable speed exercise treadmill (Eco 3/6 Treadmill, Columbus Instruments, Ohio, USA) as follows: following a 5 min warm-up period at 10 m/min, the speed of the treadmill was increased by 1 m/min every 2 min up to 45 min with no further increase in speed beyond 30 m/min. Exhaustion was defined as the point where mice were unable to avoid low-intensity electrical stimulation for 10 s by running on the treadmill track. At this time mice were generally breathing heavily and had difficulty righting themselves when placed on their backs. Endurance exercise training consisted of a 5 min warm-up as above and then a 30 min main bout (12 m/min). The main bout was increased by 2 m/min every 2 weeks until 16-18 m/min and then the duration was increased to 45 min.

### Muscle Fiber Permeabilization and Mitochondrial Respiration

A portion of freshly isolated *quadriceps femoris* muscle was placed into ice-cold permeabilization solution (10 mM Ca^2+^/EGTA buffer, 0.1 µM free calcium, 5.77 mM Na_2_ATP, 6.56 mM MgCl_2_, 20 mM Taurine, 15 mM Na_2_Phosphocreatine, 20 mM Imidazole, 0.5 mM DTT, and 50 mM MES; BIOPS) to isolate fiber bundles as described [64]. Muscle was carefully dissected free of any fat and connective tissue and separated into bundles under a dissection microscope with fine forceps for 20 minutes. Fibers were then collected, immersed in fresh BIOPS solution supplemented with saponin (50 µg/ml) and incubated at 4°C on a rotator for 30 mins. Bundles were then washed twice in respiration buffer (0.5 mM EGTA, 3 mM MgCl_2_, 60 mM K-lactobionate, 20 mM taurine, 10 mM KH_2_PO_4_, 20 mM HEPES, 110 mM sucrose, and 1 g/L BSA (fatty acid free), pH 7.1) for 5 mins on a rotator at 4°C to remove any residual permeabilization solution. Permeabilized fiber bundles were blotted dry, weighed (1–2 mg) and transferred to a high-resolution respirometer (Oxygraph-2k, Oroboros Instruments, Innsbruck, Austria) containing air saturated respiration buffer at 37°C and the chamber was closed. Standardized calibrations to correct for background oxygen flux were completed prior to performing the experimental procedures. All experiments were performed in duplicate, simultaneously.

Resting, basal respiration without adenylates was obtained by the addition of 10 mM glutamate (G) and 2 mM malate (M) as substrates of complex I (a surrogate for State 4 in permeabilized fibers). This was followed by injection of 2.5 mM ADP (D) to assess oxidative phosphorylation through complex I with maximal ADP stimulation (State 3). The subsequent addition of succinate (10 mM, S) provided the measurement of convergent electron flux through complexes I and II. Mitochondrial outer membrane intactness was tested by the addition of 10 µm cytochrome c after ADP addition; no change in respiration was observed in our preparations in the presence of cytochrome c. We also calculated the respiratory control ratio (RCR  =  State 3/State 4) as the ratio of respiration with glutamate and malate with and without ADP, respectively. Oxygen consumption measurements were normalized to tissue weight for all substrate combinations. Experiments were performed at oxygen concentrations greater than 80 µM to prevent diffusion limitations present in permeabilized fibers. Measurements were acquired using steady state regions of oxygen flux following substrate addition.

### Protein Analysis

Frozen muscle tissue (*tibialis anterior*) was homogenized in 20 vol/mass NP40 lysis buffer (50 mM Tris, 150 mM NaCl, 1% NP40, pH 7.4) supplemented with protease inhibitor cocktail (#11873580001, Roche Diagnostics, QC, Canada) and phosphatase inhibitor (PhosSTOP, #04906837001, Roche Diagnostics, QC, Canada). Protein concentrations were determined using Pierce® BCA Protein Assay Kit (#23227, Thermo Scientific, IL, USA). Denatured proteins were separated using SDS-PAGE and the expression of specific proteins analyzed using immuno-blotting (Western blotting). Anti-VDAC (#4866), -p38 MAPK (#9212), -phospho-p38 MAPK (Thr180/Tyr182) (#9215), -AMPK (#2532), -phospho-AMPK (#2531), -LDHA (#2012), -α/β-Tubulin (#2148), -p44/42 MAPK (ERK1/2) (#9102), and JNK (#9258) antibodies were obtained from Cell Signaling Technology (MA, USA). Anti-CytC (#556433, BD Pharmingen, NJ, USA), and -actin (#612657, BD Transduction Labs, NJ, USA), -PGC-1α (#516557, Millipore, MA, USA), -phospho-JNK1/2 (Thr183/Tyr185) (#44682G, Invitrogen, ON, Canada), -phospho-ERK (Tyr204) (#sc-7383, Santa Cruz Biotechnology Inc., CA, USA), was also used. MitoProfile® Total OXPHOS Rodent WB Antibody Cocktail (#MS604, Mitosciences, OR, USA) was used to detect mitochondrial proteins in Complex I, II, III, IV, & V. OxyBlot^TM^ Protein Oxidation Detection Kit (#S7150, Millipore) was used to detect protein carbonyls. Specific immuno-reactive bands were quantified using ImageJ software (http://rsbweb.nih.gov/ij/docs/index.html, NIH, USA).

### Statistical Analyses

All of the aforementioned anthropometric measurements and molecular indices between the groups were analyzed by two-way ANOVA using Prism 5 software (GraphPad Software Inc., CA, USA). Where appropriate post-hoc two-tailed T-Tests (Bonferroni corrected) were conducted to analyze differences between antioxidant supplemented and placebo supplemented groups. Statistical significance was established at P ≤ 0.05. Data are presented as mean ± SEM.

Methods for RNA analysis are provided in the supporting information (Methods S1, Table S1).

## Supporting Information

Figure S1 Expression of mitochondrial proteins and Tfam. Expression of specific subunits within complex III and IV of mitochondrial electron transport chain along with Tfam were determined as in Figure 3. φ indicates main effect of training, p≤0.05, and post-hoc testing was used to compare antioxidant- and placebo-supplemented groups, * indicates p≤0.05.
(TIFF)

Figure S2 Expression of mitochondrial proteins. Expression of specific subunits within complex I, II, and V of the mitochondrial electron transport chain were determined as in Figure 3. φ indicates main effect of training, p≤0.05, and post-hoc testing was used to compare antioxidant- and placebo-supplemented groups, * indicates p≤0.05.
(TIFF)

Figure S3 Expression of antioxidant defense enzymes. Expression of the antioxidant enzymes MnSOD, Cu/ZnSOD, and catalase were determined as in Figure 3. φ indicates main effect of training, p≤0.05, and post-hoc testing was used to compare antioxidant- and placebo-supplemented groups, * indicates p≤0.05.
(TIFF)

Figure S4 Activation of stress kinases. Protein expression of total-p44/42 MAPK (ERK1/2), phospho-p44/42 MAPK (phospho-ERK1/2, Tyr204), total-JNK1/2, and phospho-JNK1/2 (Thr183/Tyr185) was determined as in Figure 3. The ratio of phosphorylated bands to total bands was determined and plotted. φ indicates a significant (p≤0.05) main effect of exercise and post-hoc testing was used to compare antioxidant- and placebo-supplemented groups, * indicates p≤0.05.
(TIFF)

Figure S5 Food intake and body weight. Average daily food intake was calculated from weekly measurements of food weight. Food intake was unaffected by either antioxidant supplementation or training in both males and females. Whole body weight measurements were performed prior to animal sacrifice. In females, body weight was unaffected by antioxidant supplementation or training. However, in males, there was a significant main effect (p = 0.0123) for training where trained males had reduced body weight. φ indicates a significant (p≤0.05) main effect of exercise.
(TIFF)

Figure S6 mRNA expression of NFκB target genes. Quantitative RT-PCR was conducted on mRNA extracted from *tibialis anterior* muscle tissue. Genes that are specifically activated via the NFκB pathway were selected for examination. mRNA expression of *Cyclooxenase 2*, *sod1*, and *sod2* was not significantly affected by antioxidant supplementation, acute exercise, or training. This suggests that the NFκB pathway may not play an important role in the mechanism of improved performance and mitochondrial function by antioxidant supplementation in these mice.
(TIFF)

Figure S7 Respiratory capacity was assessed in permeabilized fibers from *quadriceps femoris* muscle tissue (n = 3–4). Basal respiration (State 4 surrogate) was measured in the presence of complex I substrates (glutamate and malate, upper panels) and ADP was added to measure State 3 respiration. RcR was calculated as the ratio between State 3 and State 4 respiration (G+M+D / G+M, lower panels). There were no significant differences in State 4 respiration regardless of antioxidant or training status. There was a significant interaction between antioxidant supplementation and training with respect to RcR (p≤0.05), and post-hoc testing was used to compare antioxidant- and placebo-supplemented groups, * indicates p≤0.05. The effect of training (alone) on these measures is shown in the inset figure (dotted border) (unpaired T-Test, ** indicates p<0.01).
(TIFF)

Table S1 Primer Sequences. Primer sequences used in RT-PCR analyses of the expression of specific genes.
(DOC)

Methods S1 (DOC)
