# Supplementary material for: Supplementation with α-Lipoic Acid, CoQ10, and Vitamin E Augments Running Performance and Mitochondrial Function in Female Mice
Source: PLoS One. 2013 Apr 2;8(4):e60722. doi: 10.1371/journal.pone.0060722 (PMC3614986; doi:10.1371/journal.pone.0060722)
Supplement: Table S1 — Primer Sequences. Primer sequences used in RT-PCR analyses of the expression of specific genes. (DOC) [file pone.0060722.s008.doc]

**Table S1:**

| **Gene** | **Forward primer** | **Reverse primer** |
| --- | --- | --- |
| PS2 (cyclooxegenase) | cacaacagagtgtgcgacata | gctcaggtgttgcacgtagt |
| SOD1 | ggttccacgtccatcaagta | acgtcctttccagcagtc |
| SOD2 | caagcgtgactttgggtctt | tgcaatgggtcctgattaga |
