# Supplementary material for: Supplementation with α-Lipoic Acid, CoQ10, and Vitamin E Augments Running Performance and Mitochondrial Function in Female Mice
Source: PLoS One. 2013 Apr 2;8(4):e60722. doi: 10.1371/journal.pone.0060722 (PMC3614986; doi:10.1371/journal.pone.0060722)
Supplement: Methods S1 — (DOC) [file pone.0060722.s009.doc]

**Supplemental Methods:**

***RNA Analysis:*** Total RNA was extracted from 15-20 mg of muscle (*tibialis anterior*) tissue using a Qiagen RNeasy Mini Kit (Qiagen, CA, USA) in accordance with the manufacturer’s instructions. RNA integrity and concentration was assessed using the Agilent 2100 bioanalyzer. First-strand cDNA synthesis from 1 μg of total RNA was performed with random primers using a high-capacity cDNA reverse-transcription kit (Applied Biosystems, CA, USA) in accordance with the manufacturer’s directions. RT-PCR procedures were followed as previously described , and specific primer sequences are presented in Table S1. β2 microglobulin was used as a control gene because its expression was not affected by the experimental interventions.

**references:**

1. Safdar A*, et al.* (2011) Endurance exercise rescues progeroid aging and induces systemic mitochondrial rejuvenation in mtDNA mutator mice. *Proc Natl Acad Sci U S A* 108(10):4135-4140.

2. Safdar A, Abadi A, Akhtar M, Hettinga BP, & Tarnopolsky MA (2009) miRNA in the regulation of skeletal muscle adaptation to acute endurance exercise in C57Bl/6J male mice. *PLoS One* 4(5):e5610.
